# Editorial: Biomarkers and beyond: predicting course and tailoring treatment in inflammatory bowel diseases

**DOI:** 10.3389/fimmu.2026.1898347

**Published:** 2026-06-17

**Authors:** Arianna Dal Buono, Olga Maria Nardone, Rocio Ferreiro-Iglesias

**Affiliations:** 1 IBD Center, IRCCS Humanitas Research Hospital, Rozzano, Milan, Italy; 2 Department of Biomedical Sciences, Humanitas University, Pieve Emanuele, Milan, Italy; 3 Gastroenterology, Department of Public Health, University of Naples Federico II, Naples, Italy; 4 Gastroenterology Department, Hospital Clinico Universitario de Santiago de Compostela, Santiago de Compostela, Spain; 5 Fundacion Instituto de Investigacion Sanitaria de Santiago (IDIS), Santiago de Compostela, Spain

**Keywords:** biomarkers, disease prediction, disease progression, inflammatory bowel disease, multi-omcis

Inflammatory bowel diseases (IBD) are highly heterogeneous disorders characterized by variable disease course, therapeutic response, and long-term outcomes. In this Research Topic, *“*^1^*”*, investigators explored emerging biomarkers, multi-omic approaches, and artificial intelligence (AI)-driven strategies aimed at advancing precision medicine and individualized care in IBD.

Despite major therapeutic advances, clinicians still face substantial uncertainty when attempting to predict disease progression, therapeutic response, and long-term outcomes in individual patients with IBD. Current management strategies remain largely reactive and phenotype-driven, highlighting the urgent need for biologically informed tools capable of supporting earlier and more personalized intervention.

## 1 Biomarkers for diagnosis and prognosis

The identification of reliable biomarkers for diagnosis and prognosis remains one of the major unmet needs in IBD. Several contributions within this Research Topic explored novel molecular and serological markers capable of improving disease detection and characterization beyond conventional inflammatory indices.

Among the most clinically relevant findings, Bez et al. demonstrated that antibodies targeting integrin αvβ6 show high diagnostic accuracy for ulcerative colitis, supporting their potential role as non-invasive biomarkers for earlier diagnosis. Similarly, Zhang et al. evaluated the triglyceride-glucose index in a large UK Biobank cohort, identifying metabolic alterations associated with increased IBD risk and reinforcing the relevance of systemic metabolic pathways in disease pathogenesis.

Beyond diagnosis, several studies focused on biomarkers associated with disease progression and prognosis. Tan et al. provided a systematic review and meta-analysis on peripheral inflammatory markers such as neutrophil-to-lymphocyte ratio (NLR), platelet-to-lymphocyte ratio (PLR), and lymphocyte-to-monocyte ratio (LMR), highlighting their potential utility as accessible prognostic tools in both Crohn’s disease and ulcerative colitis. In parallel, Feng et al. explored the contribution of vitamin D deficiency and modifiable risk factors in Crohn’s disease, emphasizing the growing interest in biomarkers reflecting nutritional, environmental, and metabolic determinants of disease activity.

Collectively, these studies illustrate the ongoing transition from isolated inflammatory markers toward multidimensional biomarker models integrating immunologic, metabolic, and clinical information. Although validation and standardization remain necessary before routine implementation, these approaches may contribute to earlier diagnosis, improved risk stratification, and more individualized monitoring strategies in IBD.

## 2 Prediction of disease course and treatment response

Predicting disease evolution and therapeutic response remains one of the central challenges in precision medicine in IBD. Several articles included in this Research Topic investigated biomarkers associated with disease progression, treatment efficacy, and long-term clinical outcomes, with the aim of facilitating more proactive and personalized management strategies. A particularly relevant contribution came from Polamarasetty et al., which showed that baseline stool TIMP-2 levels may predict stricturing and penetrating disease progression in Crohn’s disease. This finding highlights the potential role of non-invasive biomarkers in identifying patients at risk of developing complicated disease phenotypes before irreversible bowel damage occurs. Similarly, Wu et al. explored transcriptomic and single-cell analyses of ileal tissue, identifying molecular pathways associated with hypercoagulability and disease severity in Crohn’s disease. These data support the concept that coagulation and inflammatory pathways may interact in driving disease progression and could serve as potential therapeutic targets.

Therapeutic response prediction also emerged as a main focus within this Research Topic. Wang et al. applied multi-omic analyses to identify efferocytosis-related hub genes associated with ustekinumab response in colitis, illustrating how integrated molecular profiling may help optimize biologic selection. Furthermore, Gimeno-Pitarch et al. evaluated biomarker dynamics during intravenous-to-subcutaneous vedolizumab transition in refractory pouchitis, providing insights into real-world therapeutic monitoring and treatment adaptation.

Together, these studies reinforce the growing shift from reactive disease management toward predictive and biologically informed strategies. The integration of prognostic biomarkers with therapeutic response signatures may ultimately support earlier intervention, optimized treatment sequencing, and more individualized long-term care in IBD.

## 3 Integration of multi-omics and machine learning

The increasing integration of multi-omic technologies and AI-driven analytics is progressively transforming translational research in IBD. By combining transcriptomics, immunology, clinical phenotyping, and computational modelling, these approaches aim to improve disease stratification, identify new therapeutic targets, and support precision medicine frameworks.

Several studies within this Research Topic exemplify this evolving paradigm. Calviño-Suárez et al. investigated sex-dependent activation of the JAK2/STAT3 pathway in ulcerative colitis, demonstrating how transcriptomic analyses may uncover biologically distinct inflammatory signatures associated with disease heterogeneity. Similarly, Wang et al. integrated multi-omic datasets to identify efferocytosis-related biomarkers predictive of ustekinumab response, highlighting the growing role of systems biology approaches in therapeutic personalization.

Digital medicine and AI were further explored in the review by De Deo et al., which discussed the emerging role of digital biomarkers, machine learning algorithms, and real-world data integration in personalized IBD management. The authors emphasized the potential of AI-driven models to facilitate interpretation of complex biomarker datasets, improve predictive analytics, and support dynamic clinical decision-making.

At the same time, these advances raise important challenges regarding data standardization, reproducibility, interpretability, and external validation. While multi-omics and AI remain at an early stage of clinical implementation, the studies included in this Research Topic highlight their growing potential to move beyond traditional phenotype-based classifications toward a more biologically driven understanding of IBD. Such approaches may ultimately support more precise prediction of disease behaviour, therapeutic response, and long-term outcomes.

## 4 Clinical translation and future perspectives

The rapid expansion of biomarker research in IBD is progressively bridging the gap between biological discovery and clinical application. Although many biomarkers remain exploratory, increasing efforts are focused on identifying clinically actionable tools able to support diagnosis, risk stratification, therapeutic decision-making, and longitudinal disease monitoring within precision medicine frameworks.

One of the major advances in translational medicine is the shift from descriptive associations toward mechanistically informed biomarkers. Transcriptomic and molecular profiling technologies are increasingly identifying biological pathways associated with disease progression and immune dysregulation, supporting a transition from phenotype-based classifications toward biologically driven disease characterization. Recent studies have highlighted mechanisms linked to hypercoagulability in Crohn’s disease (Wu et al.), and Calviño-Suárez et al., illustrating how molecular profiling may contribute to a better understanding of disease heterogeneity and progression.

At the same time, growing attention is being directed toward non-invasive and clinically applicable biomarkers. Blood-based transcriptomic signatures for IBD diagnosis (Nazari et al.) and antibodies targeting integrin αvβ6 with high diagnostic accuracy for ulcerative colitis (Bez et al.) represent promising alternatives to invasive procedures. Similarly, biomarkers associated with disease progression, such as stool TIMP-2 for predicting stricturing and penetrating Crohn’s disease behaviour (Polamarasetty et al.) may support earlier risk stratification and more proactive disease management. The identification of molecular alterations during preclinical phases of Crohn’s disease (Kioroglou et al.) further reinforces the possibility of earlier diagnosis and intervention, potentially improving long-term outcomes. Integrated inflammatory and coagulation biomarker models may also contribute to surgical risk assessment and therapeutic decision-making in Crohn’s disease (Wu et al.).

Artificial intelligence (AI) and digital technologies are also emerging as important components of translational medicine in IBD. By integrating molecular, clinical, endoscopic, imaging, and real-world data, AI-driven approaches may facilitate the interpretation of complex biomarker datasets, support predictive analytics, and enable more refined patient stratification (De Deo et al.). However, robust validation, standardization, transparency, and clinical interpretability remain essential before widespread implementation can be achieved.

Despite the growing number of candidate biomarkers and multi-omic approaches, no sufficiently validated and reproducible tools are currently available in routine clinical practice to reliably individualize therapy or accurately predict disease course at the individual patient level. Nevertheless, ongoing advances in biomarkers, multi-omics, and AI are progressively paving the way toward a more proactive, dynamic, and biologically informed model of personalized IBD care.
